# Risk of HBV transmission from HBcAb-positive grafts in pediatric liver transplantation: a real-world prospective cohort study

**DOI:** 10.1186/s12985-025-02941-1

**Published:** 2025-09-29

**Authors:** Yuting Yang, Guojin Wu, Xiaoke Dai, Tengteng Zhang, Bingqian Tan, Yue Lu, Mingman Zhang, Yao Zhao

**Affiliations:** 1 National Clinical Research Center for Child Health and Disorders, Ministry of Education Key Laboratory of Child Development and Disorders, Chongqing Key Laboratory of Pediatric Metabolism and Inflammatory Diseases, Children’s Hospital of Chongqing Medical University, Chongqing, China; 2 Department of Hepatobiliary Surgery, Children’s Hospital of Chongqing Medical University, Chongqing, China; 3 Biobank Center of Children’s Hospital of Chongqing Medical University, Chongqing, China

**Keywords:** Pediatric recipient, Liver transplant, HBcAb-positive grafts, Occult HBV infection, HBV transmission

## Abstract

**Background:**

The utilization of liver grafts from hepatitis B core antibody (HBcAb)-positive donors is relatively common in regions with high hepatitis B virus (HBV) prevalence. This practice poses a potential risk of HBV transmission. However, the impact of these grafts on pediatric liver transplant recipients is not well-established.

**Method:**

To address these knowledge gaps, we conducted a prospective observational cohort study to assess the risk of post-transplant HBV transmission in pediatric recipients of HBcAb-positive grafts. Hepatitis B serology and liver tissue analyses for HBV DNA were performed during post-transplantation follow-up of 188 pediatric recipients.

**Results:**

In the cohort study, 43 pediatric recipients (22.9%) received HBcAb-positive grafts, while 145 (77.1%) received HBcAb-negative grafts. Over a median follow-up of 43 weeks, 10 recipients (5.3% of the total cohort) developed HBV infection. The cumulative incidence of de novo HBV infection was significantly higher in recipients of HBcAb-positive grafts (18%, 95% CI: 5–31) compared to recipients of HBcAb-negative grafts (3%, 95% CI: 0–6, *p* < 0.05). Notably, higher levels of hepatitis B surface antibody (HBsAb ≥ 100mIU/ml) in pediatric recipients were associated with a significantly reduced risk of post-transplant de novo HBV infection (*p* < 0.05).

**Conclusion:**

HBcAb-positive grafts substantially increase HBV transmission risk in pediatric liver transplantation. Elevated HBsAb titers may mitigate infection severity, while occult HBV infections require vigilant monitoring. Strategic enhancement of recipient HBsAb levels and optimized prophylactic protocols are critical for improving outcomes.

**Supplementary Information:**

The online version contains supplementary material available at 10.1186/s12985-025-02941-1.

## Introduction

Liver transplantation is recognized as the most effective treatment of choice for patients with acute and chronic end-stage liver disease, selected liver tumors, and certain metabolic disorders [1]. Due to the persistent scarcity of donor organs, the utilization of grafts that are from donors with hepatitis B core antibody (HBcAb) is rather common [2–4]. However, this practice raises significant concerns regarding the potential for hepatitis B virus (HBV) transmission to recipients. While studies in adult populations have demonstrated the risk of de novo HBV infection in susceptible recipients receiving grafts from HBsAg-negative/HBcAb-positive donors [5, 6] the implications of utilizing such grafts in pediatric liver transplantation remain less well-defined. Given that liver transplantation is a critical therapeutic modality for infants and children with liver failure and metabolic disease [7, 8] the impact of HBcAb-positive grafts on the incidence of de novo HBV infection in this vulnerable population warrants further investigation, as current evidence regarding their safety and efficacy in pediatric recipients is limited.

It is crucial to recognize that the clinical efficacy of liver transplantation in pediatric patients may be substantially compromised by the occurrence of HBV infection post-transplantation. The chronicity of HBV infection during early childhood can reach up to 80% [9], which may pose the substantial burdens due to liver-related morbidity and mortality [10]. To effectively mitigate the potential consequences of HBV infection in children following liver transplantation, it is essential to investigate the risk of de novo HBV infection in pediatric recipients of HBcAb-positive liver grafts. In the present study, we conducted a prospective study to assess the risk of de novo HBV infection occurrence in pediatric patients receiving liver grafts from HBcAb-positive donors, and to explore potiential protective factors.

## Methods

### Pediatric liver transplant recipients and study design

This was a real-world prospective study that conducted in the National Clinical Research Center for Child Health and Disorders of China. Our center is the unique children’s hospital with liver transplant qualification in China, and had constructed the first pediatric liver transplantation biobank since 2018 [11], which can effectively provide the shortage of resources in scientific research on pediatric liver transplantation. Consecutive HBsAg-negative, pediatric liver transplantation patients younger than age 12 years, with newly diagnosed a pediatric end-stage liver disease (e.g. cholestatic liver disease, inborn errors of metabolism, etc.), were recruited between September 2018 and September 2023. Exclusion criteria included HBsAg and(or) HBV DNA positive before liver transplantation; patients with no post-transplantation follow-up data on their HBV serological status; donation of citizen’s death (DCD) liver donors. Data on the hepatitis B vaccination status of all pediatric recipients, including the number of doses received prior to transplantation, were collected from medical records where available. Information regarding any specific anti-HBV prophylactic measures, such as the use of hepatitis B immunoglobulin (HBIG) or nucleos(t)ide analogues (NAs), was also collected.

After liver transplantation, all pediatric recipients will undergo clinical follow-up to monitor hepatitis B serological markers and (or) perform HBV DNA testing. The main study end point was de novo HBV infection post liver transplantation, which was defined as HBsAg seropositivity and (or) HBV DNA positive.

The study was approved by the Ethics Review Committee of the Children’s Hospital of Chongqing Medical University (No. 2019-24), and register on clinical trial.gov (NCT03865966).

### Follow-up and clinical HBV virological monitoring

The recommended clinical follow-up schedule involved visits every 2 weeks for the first 2–3 months, progressively extended to every 1–3 months up to 24 months, and every 5–6 months thereafter. HBV status was monitored during these visits. As a real-world study, the actual frequency of HBV virological testing varied among patients, reflecting routine clinical practice and depending on factors such as clinical stability, guardian compliance, and socioeconomic considerations.

HBV serology were measured before the initiation of liver transplantation and during the follow-up using chemiluminescence microparticle immunoassay (CMIA) kits (Abbott GmbH & Co. KG, Wiesbaden, Germany). Subjects were considered HBsAg-positive at values ≥ 0.05 IU/mL, HBsAb-positive, or seroprotected at values ≥ 10 mIU/mL, HBeAg-positive at a sample rate/cut off rate ≥ 1 S/CO, HBeAb-positive at values ≤ 1 S/CO, and HBcAb-positive at values ≥ 1 S/CO. HBV DNA was detected by a quantitative diagnostic kit with a limit of quantitation (LOQ) of 400 IU/mL (Shengxiang, Hunan, China).

### Quantitation of intrahepatic HBV total DNA and CccDNA by DdPCR

Intrahepatic total DNA was extracted from pediatric receptors’ frozen liver tissue using QIAamp DNA kit (QIAGEN Inc., Germany) according to the manufacturer’s instructions, and concentration was assessed by NanoDrop ND 1000 (NanoDrop Technologies, Wilmington, Delaware, USA). GAPDH gene detection with a real-time quantitative PCR was used to evaluate the quality of hepatic DNA extracted.

The primers and probes for HBV total DNA and covalently closed circular (cccDNA) detection were displaying in Supplementary Table 1. HBV cccDNA detection method used was referenced from Caviglia et al. [12]. For HBV cccDNA quantitation in hepatic DNA extracts, intrahepatic total DNA were treated 30 min at 37℃ with 10U of plasmid-safe ATP dependent DNase (PSAD) (Epicentre, Madison, Wisconsin, USA) to digest single-strand DNA and linear double-strand DNA. A detailed description of the droplet digital PCR (ddPCR) method for the detection of HBV total DNA and cccDNA can be found in the supplementary methods.

### HBV fragment amplifying and sequencing by nested PCR

Extracted hepatic DNA samples were also analyzed for the presence of HBV genomes by four parallel nested PCRs to detect HBV surface, core, polymerase and X sequences. PCR primers were complementary to highly conserved nucleotide sequences of HBV genome [13]. The PCR mix was the same for all reactions and comprised Taq PCR Master Mix (Tiagen, Beijing, China), the first and the second-round primers, and H_2_O. Primary HBV DNA were used in the first round PCR, and the first round PCR product was used as the template for the second round. Amplification was performed for 35 cycles of denaturation at 94 ℃ for 30 s, annealing at 55 ℃ for 30 s, and elongation at 72 ℃ for 1 min, followed by a final extension at 72 ℃ for 5 min. Finally, the second round of PCR product was analyzed by electrophoresis in agarose gel, and positive fragments were sequenced.

### Immunosuppressive protocol

The immunosuppressive protocol was standardized based on the national guidelines for liver transplantation [14]. The regimen consisted of induction and maintenance therapy. Induction therapy utilized the interleukin-2 receptor antagonist, basiliximab, which was administered pre-operatively and on post-operative day 4, with dosing based on standard weight-based recommendations. Maintenance therapy was centered on a tacrolimus-based regimen. Dosing was individually adjusted to achieve target whole-blood trough concentrations, guided by the patient’s clinical course and the time elapsed since transplantation. This regimen was combined with corticosteroids, which were initiated intravenously and subsequently tapered; steroid withdrawal was generally completed by 3 to 4 months post-transplantation. In cases where the initial regimen of tacrolimus and corticosteroids required modification, adjunctive agents such as mycophenolate mofetil (MMF) were introduced. The use and dosing of such agents were tailored to individual patient needs to optimize efficacy or manage side effects.

### Statistical analyses

Continuous variables were expressed as median (interquartile range). The Mann-Whitney non-parametric test was used for comparison of continuous variables, and Fisher’s exact test or chi-square (χ2) test was used for comparing categorical variables. The cumulative rate of de novo HBV infection was estimated by the Kaplan-Meier method. The log-rank test compared risk factors for de novo HBV infection post-liver transplantation, defining outcome events as de novo HBV infection. The Kaplan-Meier method estimated the cumulative rate of de novo HBV infection. The Cox proportional hazards model tested the hazard ratio for HBV reactivation. Univariable analysis considered factors like age, sex, baseline HBsAb levels, HBsAb status (< 100 v ≥ 100mIU/ml), receptors’ HBcAb status (positive v negative), donor source, and donors’ HBcAb status, and factors with *P* < 0.10 were included in a multivariable Cox model. All statistical analyses were performed using SPSS version 19.0 (SPSS, Chicago, IL). A two-sided *P* ≤ 0.05 was considered statistically significant.

## Results

### Pediatric patients included and baseline characteristics

**Fig. 1:**
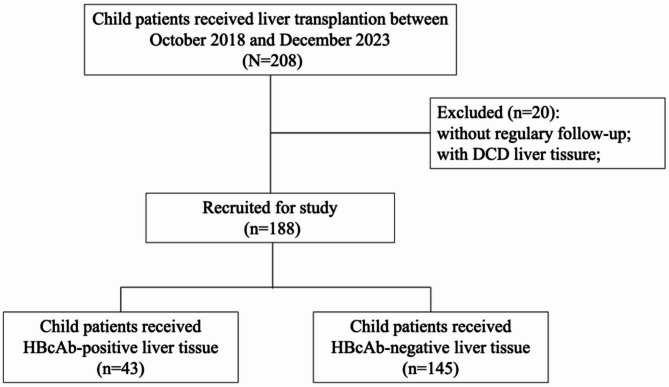
Flow chart depicting patient deposition

A prospective observational cohort study was conducted, 208 pediatric patients received liver transplantation during the study period from the year of 2018 to 2023. After excluding 7 patients who had irregularly follow-up and 13 were receiving DCD donors, 188 patients were recruited based on previously defined inclusion and exclusion criteria (Fig. 1; Table 1), of whom 43 (22.9%) and 145 (77.1%) were received HBcAb-positive and negative liver grafts respectively. The overall pre-transplant HBcAb positivity rate in recipients was 22.3% (42/188). This rate was significantly higher in children scheduled to receive an HBcAb-positive graft compared to those receiving an HBcAb-negative graft (60.4% [26/43] vs. 11.0% [16/145], *P* < 0.001). The median duration of follow-up was 105 weeks (range, 76 to 139 weeks) and 89.7 weeks (range, 30 to 153 weeks) for the two groups, and all included pediatric patients alive well at the end of follow-up. In line with national policy, nearly all patients had received the initial dose of the hepatitis B vaccination. But the standard vaccination schedule was commonly disrupted by their underlying clinical condition. Consequently, it was estimated that less than half of the patients (approx. 40%) received a second dose, while completion of the full three-dose series prior to transplantation was rare (approx. 1%). Beyond this incomplete vaccination, specific anti-HBV prophylaxis was uncommon, and only a small portion of children received HBIG or other combined measures around the time of transplantation.

**Table 1:** The characteristics of pediatric patients transplant with HBcAb-positive and HBcAb-negative liver tissue

	With HBcAb-positive grafts	With HBcAb-negative grafts
Number	43	145
Age (month)	5 (4, 6)	5 (5, 6)
Sex (F/M)	15/28	75/70
HBsAg-positive (%)	0 (0%)	0 (0%)
HBsAb level		
Titer, mIU/ml	137.7 (27.7, 330.1)	144.9 (38.9, 616.9)
[0, 100), %	20 (46.5%)	59 (40.7%)
[100, 500), %	15 (34.9%)	45 (31.0%)
≥ 500, %	8 (18.6%)	41 (28.3%)
HBcAb positive rate	26 (60.4%)	16 (11.0%)
Source of donor (mother/father)	29/14	84/61
Duration of follow-up (weeks)	105 (76, 139)	89.7 (30.4, 153.1)

### Occurrence of de Novo HBV infection in pediatric patients after liver transplantation

**Fig. 2:**
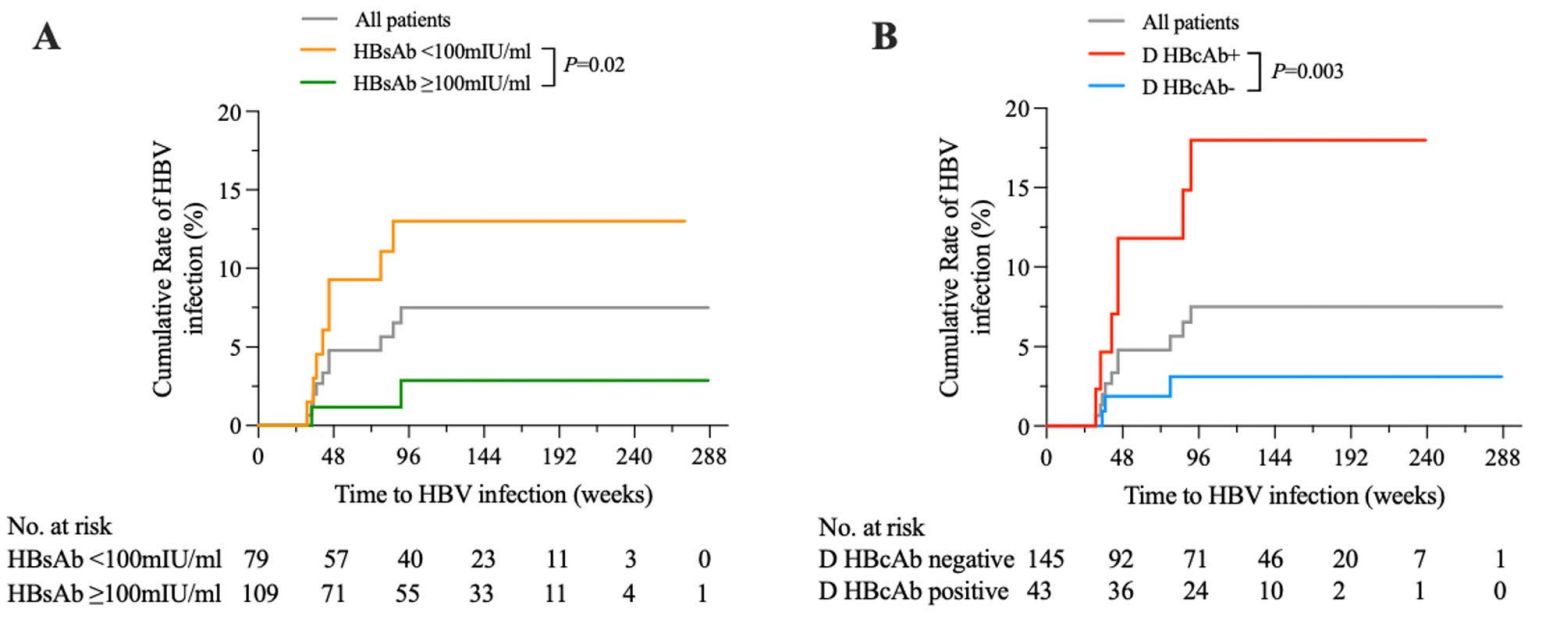
Cumulative rate of de novo HBV infection after liver transplantation based on different serological status. (**A**) Kaplan-Meier curve showing the cumulative incidence of de novo HBV infection in recipients stratified by their pre-transplant baseline levels of HBsAb. (**B**) Kaplan-Meier curve showing the cumulative incidence of de novo HBV infection based on the donors’ HBcAb status

**Fig. 3:**
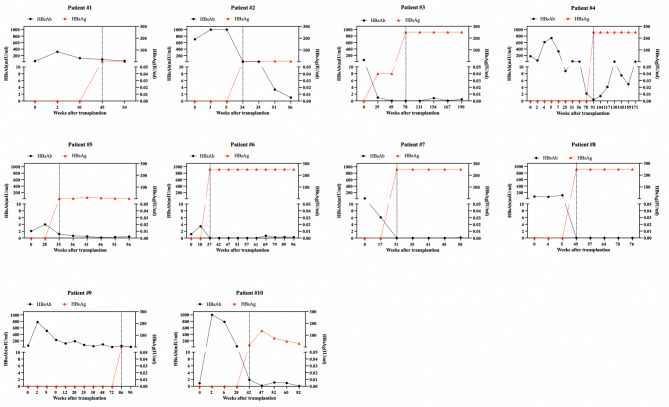
Changes in HBsAg and HBsAb in children who developed HBV infection

Out of the 188 children with tests for HBV seromarkers after liver transplantation, 10 pediatric recipients (10/188, 5.3%) were found to have developed HBV infection after liver transplantation with continuous HBsAg positivity, at the median of 43 weeks (range, 35.6 to 70 weeks). The accumulative rates of de novo HBV infection at 1 year and 2 years were 5% and 7.5%, respectively (Fig. 2). The changes of HBV seromarkers and details of pediatric patients with de novo HBV infection were shown in Table 2; Fig. 3. As illustrated by the individual patient trajectories in Fig. 3, a progressive decline in post-transplant HBsAb levels was observed in all of these patients prior to the detection of HBV infection. Two pediatric patients had low levels of HBV load (below the detection limit, < 400 IU/ml) at the time of HBsAg seroreversion. Nine pediatric patients were younger than 1 year old when transplanted with liver tissue because of biliary atresia, and one patient was 10 years old with hepatolenticular degeneration. With respect to the status of HBcAb in donors, 7 pediatric patients transplanted with HBcAb-positive liver grafts.

**Table 2:** Details of 10 pediatric patients with HBV infection after liver transplantation

	Receptor baseline					Donor			Receptor HBV infection^#^							Outcome
Patient	Sex	Age (months)	HBsAg	HBsAb (mIU/ml)	HBcAb	HBsAg	HBsAb (mIU/ml)	HBcAb	Weeks to infection	HBV DNA	HBsAg (IU/ml)	HBsAb (mIU/ml)	HBeAg	HBeAb	HBcAb	
1	M	7	Neg	31.3	Neg	Neg	33.0	Pos	45	1.93 × 10^3^	5.42	82.7	Pos	Neg	Neg	Alive and well
2	M	5	Neg	702.0	Pos	Neg	16.4	Pos	34	< 400	6.34	3.35	Neg	Neg	Neg	Alive and well
3	F	6	Neg	63.5	Neg	Neg	119.2	Neg	78	> 1 × 10^8^	> 250.0	0	Pos	Neg	Neg	Alive and well
4	F	5	Neg	177.2	Neg	Neg	249.8	Pos	91	3.8 × 10^4^	> 250.0	0.38	Pos	Neg	Neg	Alive and well
5	M	5	Neg	2.06	Neg	Neg	282.4	Neg	35	2.8 × 10^4^	2.24	1.2	Pos	Neg	Neg	Alive and well
6	F	120	Neg	0.2	Neg	Neg	5.03	Neg	37	1.77 × 10^8^	> 250.0	0	Pos	Neg	Neg	Alive and well
7	M	5	Neg	22.5	Pos	Neg	311	Pos	31	1.38 × 10^5^	> 250.0	0	Pos	Neg	Neg	Alive and well
8	M	5	Neg	75.3	Pos	Neg	20.1	Pos	45	6.74 × 10^5^	> 250.0	0	Pos	Neg	Neg	Alive and well
9	F	4	Neg	52.16	Pos	Neg	2.2	Pos	86	< 400	4.17	15.2	Neg	Neg	Neg	Alive and well
10	M	6	Neg	0.88	Neg	Neg	595.8	Pos	41	4.8 × 10^3^	19.8	1.9	Pos	Neg	Neg	Alive and well

^**#**^: HBV status shown is at the time of initial diagnosis of de novo HBV infection during post-transplant follow-up

### Statistical analysis of the risk of de Novo HBV infection after liver transplantation

To identify independent risk factors for de novo HBV infection, we performed both univariate and multivariate logistic regression analyses. As shown in Table 3, we found that the baseline HBsAb titer of pediatric patients and HBcAb-positive donors were the important factors that associated with de novo HBV infection after liver transplantation (*p* < 0.05). The status of HBcAb in pediatric patients and source of living (mother or father) donors had no significant association with de novo HBV infection (*p* > 0.05). The cumulative rates of de novo HBV infection for the different level of HBsAb in pediatric patients were shown in Fig. 2A. Patients with HBsAb < 100mIU/ml had a significantly higher rate of de novo HBV infection than HBsAb ≥ 100mIU/ml patients (hazard ratio, 5.2; 95%CI, 1.0 to 24.4; *p* = 0.038). For HBsAb < 100mIU/ml patients, the cumulative HBV infection rates at 1 year and 2 years were 9% (95% CI: 3–15) and 13% (95% CI: 5–20), respectively. For HBsAb ≥ 100mIU/ml patients, the de novo HBV infection rates were lower, at 1% (95% CI: 0–3) and 3% (95% CI: 0–6), respectively. Furthermore, a comparison was done depending on the level of viral load when HBV infection detectable, which was negatively correlated with the HBsAb titer in the pediatric recipients after liver transplantation (Figure S1).

**Table 3:** Univariable and multivariable analyses of risk factors for de Novo HBV infection in pediatric recipients

	HBV infection	Without HBV infection	*P* (univariable)	*P* (multivariable)	HR (95%CI)
Number of recipients	10	178	-		
Duration of follow-up (weeks)	43 (35.6, 70)	103.6 (42, 152.6)	-		
Age (month)	5 (5, 6)	5 (5, 6)	0.48		
Sex (F/M)	4/6	86/92	0.75		
HBsAb Titer, mIU/ml	41.7 (7.2, 72.4)	155.4 (39.4, 577.2)	0.037		
HBsAb < 100mIU/ml, %	8 (80%)	71 (39.9%)	0.018	0.038	5.2 (1.0, 24.4)
HBcAb positive rate	4 (40%)	37 (20.8%)	0.229		
Source of donor (mother/father)	8/2	105/73	0.32		
Donors’ HBcAb positive rate	7 (70%)	36 (20%)	0.001	0.01	6.0 (1.5, 23.2)

On the effect of HBcAb status of donors (Fig. 2B), transplanting with HBcAb-positive liver tissue, when compared with HBcAb-negative donors, had a significantly higher rate of HBV infection (hazard ratio, 6; 95%CI, 1.5 to 23.2; *p* = 0.01). With HBcAb-negative donors, the cumulative HBV infection rates at 1 year and 2 years were 2% (95% CI: 0–4) and 3% (95% CI: 0–6), respectively. With HBcAb-positive donors, the accumulative rates of de novo HBV infection rates were at 12% (95% CI: 1–23) and 18% (95% CI: 5–31), which were higher than transplanted with HBcAb-negative donors.

### Analysis of pre-transplant liver tissues in patients with de Novo HBV infection

To investigate the origin of post-transplant infection, we analyzed available pre-transplant frozen liver tissues from 7 of the 10 recipients who developed de novo HBV infection using a highly sensitive ddPCR assay. The assay’s specificity was validated as shown in Figure S2. Among the seven samples tested, only one (from patient #3) was positive for both total HBV DNA (3 copies/10 µL) and cccDNA (4 copies/10 µL) (Supplementary Table 3). Notably, patient #3 was HBcAb-negative pre-transplant and received a graft from an HBcAb-negative donor. The liver tissue samples from patients #5 and #6, who also received HBcAb-negative grafts, tested negative for HBV DNA by ddPCR.

## Discussion

The clinical significance of occult HBV carriers frequently identified in individuals who are HBsAg-negative/HBcAb-positive, is well-documented [13, 15]. However, the increasing scarcity of available donors necessitates the expanded utilization of grafts from HBcAb-positive donors, particularly in regions with intermediate to high HBV endemicity. Although considerable evidence suggests that HBcAb-positive donor grafts carry a potential risk of transmission HBV infection following liver transplantation [5, 6] limited research has focused on quantifying the specific risk of de novo HBV infection in pediatric patients associated with the use of those grafts. To address those knowledge gaps, we conducted a comprehensive prospective study to specifically evaluate the risk associated with HBcAb-positive liver grafts in pediatric recipients.

In this study, we had embedded within a prospective observational cohort of living donor liver transplantations, encompassing all consecutive transplantations performed over a 6-year period in China with a relatively high prevalence of HBcAb-positive individuals in the general population. In our cohort, a total of 5.3% (10 out of 188) of pediatric patients developed HBV infection post-transplantation. Notably, the incidence of de novo HBV infection was significantly higher in children receiving HBcAb-positive grafts (16.3%, 7 out of 43) compared to those receiving HBcAb-negative grafts. This was evidenced by a 15% difference in the 2-year cumulative incidence rate of HBV activation between the two groups. The status of HBV infection in pediatric patients prior to liver transplantation remains unclear. To further ascertain whether post-transplant HBV infection results from the transmission of HBV from liver donors or the reactivation of a previous infection in recipients, liver tissue from pediatric patients was utilized as the detection sample for diagnosing occult HBV infection (OBI). Previous research has demonstrated that ddPCR offers greater sensitivity and accuracy in detecting low DNA concentrations compared to conventional PCR technologies [12]. In this study, we employed a sensitive PCR system based on ddPCR technology to analyze liver DNA extracts from recipients with de novo HBV infection. Among the seven liver samples analyzed, only one tested positive (patient #3), while the remaining samples were negative. These findings suggest that de novo HBV infection in pediatric patients following liver transplantation is primarily attributable to HBV transmission from liver grafts, with a significantly increased risk of HBV transmission to pediatric recipients when transplanting with HBcAb-positive liver grafts. It is also clinically relevant that all of these patients were negative for HBcAb at the initial diagnosis of infection. This likely reflects the early serological window of acute infection, a period prolonged by the potent immunosuppression and inherent immune immaturity of this young cohort.

A particularly challenging finding was the occurrence of de novo HBV infection in three patients (#3, #5, and #6) who were HBcAb-negative and received grafts from HBcAb-negative donors. Our analysis of their native liver tissues provides insight into these complex cases. For patient #3, the detection of both total HBV DNA and cccDNA in the pre-transplant liver tissue, despite negative serology, provides strong evidence for a pre-existing seronegative OBI in the recipient. The subsequent infection was therefore most likely due to the reactivation of this latent virus under immunosuppression. The interpretation for patients #5 and #6, whose pre-transplant liver tissues were negative by ddPCR, is more complex and highlights two critical, non-mutually exclusive possibilities. One hypothesis is that these recipients also had seronegative OBI, but the viral load was below the ddPCR detection limit or was patchily distributed within the liver, leading to a false-negative result from the sampled tissue. Alternatively, the infection could have been transmitted from the HBcAb-negative donors. It is well-documented that a small fraction of HBcAb-negative individuals can harbor seronegative OBI and act as infectious sources [16–18]. While we cannot definitively determine the origin of infection for these two patients, their cases underscore an important clinical reality: the risk of HBV transmission or reactivation is not entirely eliminated even in “HBcAb-negative to HBcAb-negative” transplant settings. This finding warrants consideration in future risk assessment and management strategies.

The estimated elevated hazard ratio for de novo HBV infection associated with transplantation from HBcAb-positive donors demonstrated temporal variability. Specifically, the risk was negligible in the early post-transplantation period and reached its peak approximately one year (43 weeks) after transplantation. This pattern contrasts with findings in adult recipients, as noted in previous studies [19]. Such a discrepancy may suggest subtle interactions between HBcAb positivity and other concurrent protective factors that enhance the recipient’s health during the first year post-transplantation. Moreover, while prior research has indicated a negative impact of HBcAb-positive liver donors on graft survival [20] we observed no graft loss among pediatric patients who received HBcAb-positive livers. It is important to highlight that all participants in this study were children, with the majority being younger than one year old. Their distinct immune status and underlying conditions may have contributed to this divergent outcome, potentially through mechanisms that merit further investigation.

A noteworthy finding was the high pre-transplant HBcAb prevalence (22.3%) in our young cohort. This observation is critical: unlike in adult transplantation where recipient HBcAb signifies past exposure and is a major risk factor for de novo HBV infection [3] in our infant cohort, it more plausibly reflects the passive transfer of maternal IgG antibodies, not their own immune history. This phenomenon is common in HBV endemic regions [21–23]. Consequently, it did not emerge as an independent predictor of transmission in our analysis. This key distinction shifts the focus of protection away from the non-prognostic HBcAb status and squarely onto the establishment of a functional immune defense, for which hepatitis B vaccination is the important. However, our study reveals a significant gap in this strategy. While nearly all infants received the first vaccination dose at birth, the progression of their underlying liver disease frequently disrupted the subsequent immunization schedule. The second dose was often delayed or omitted due to jaundice and clinical instability, and the third was commonly missed as children were undergoing or recovering from transplantation, resulting in an incomplete active immune response for most patients. This common pattern of immunization failure, coupled with the unreliability of HBcAb as a risk marker, leaves this vulnerable population in a precarious position and highlights the urgent need for optimized prophylactic strategies.

Our findings highlight a critical dilemma in pediatric liver transplantation. While current guidelines for adult recipients strongly advocate for prophylactic strategies against HBV transmission from HBcAb-positive grafts, specific, evidence-based recommendations for children remain conspicuously absent [24, 25]. This lack of guidance directly contributed to the clinical reality observed in our study: specific anti-HBV prophylaxis was applied inconsistently and infrequently. The absence of a mandated protocol meant that decisions were left to individual clinical judgment, often in a context of uncertainty regarding the true level of risk. This real-world scenario, however, does not weaken our study’s conclusions—it strengthens them. Our analysis demonstrates a significant risk of de novo HBV infection (16.3%) even within a cohort where some sporadic prophylaxis may have occurred. This underscores that incomplete pre-transplant vaccination alone is an insufficient safeguard. In this context, our data on the protective effect of high HBsAb titers (≥ 100 mIU/mL) becomes particularly valuable. The finding that recipients with higher HBsAb levels had a significantly lower infection rate (3% vs. 14%) may provide a tangible, evidence-based direction for future pediatric-specific guidelines. It suggests that maintaining robust HBsAb levels, either through a completed vaccination series or other passive immunization strategies, may be a important method in mitigating the transmission risk from HBcAb-positive grafts in children. Furthermore, the observed pattern of declining HBsAb titers preceding viral breakthrough strongly suggests that routine post-transplant monitoring of HBsAb levels could serve as a valuable clinical strategy. Such monitoring would enable timely interventions, like booster vaccinations or passive immunization, to maintain protective immunity in these high-risk patients. In addition to these immune-based strategies, the use of antiviral prophylaxis with nucleos(t)ide analogues (NAs) should be considered, particularly for the highest-risk subgroups identified in our study, such as recipients with low or undetectable baseline HBsAb titers. While NAs are the standard of care in adult settings, their optimal use in children—including patient selection, timing, and duration of therapy—requires further investigation to establish clear, evidence-based guidelines for pediatric practice.

Our study has several limitations inherent to its design. Firstly, our study is limited by its median follow–up period of two years. While this duration was sufficient to identify the peak risk of transmission within our cohort, it is not long enough to evaluate critical long-term clinical outcomes, such as the development of chronic hepatitis or graft loss. Continued follow-up of this cohort will be crucial to address these important questions. Secondly, as a real-world study, the follow-up frequency for HBV marker surveillance was not rigidly fixed, which may have led to delays in the detection of de novo infection in some cases. Thirdly, our monitoring strategy, reflective of real-world practice in many resource-limited settings, had inherent limitations. Our approach relied predominantly on serological markers, which may miss early viral replication. This was compounded by the fact that the HBV DNA assay used for follow-up had a relatively high limit of quantitation (400 IU/mL). Collectively, these factors limited our ability to detect low-level viremia and may have led to an underestimation of the true infection rate. Finally, this cohort study is constrained by its single-center design and a relatively small sample size, which may limit the statistical power and the generalizability of our findings to other populations. Despite these limitations, our study provides robust evidence that significantly contributes to the existing body of knowledge. It highlights the substantial risk of HBV transmission from HBcAb-positive grafts even under real-world surveillance conditions and underscores the necessity for developing standardized prophylactic and monitoring strategies.

In conclusion, our findings indicate that liver transplantation using HBcAb-positive grafts poses a substantial risk of de novo HBV infection in pediatric recipients. Although previous research has predominantly focused on adult populations, our study emphasizes that the clinical impact of HBcAb-positive grafts on pediatric patients warrants equal attention. In this context, the potential occurrence of seronegative OBI also remains a significant concern. Strategies designed to rapidly elevate HBsAb levels may effectively mitigate this risk. Moreover, the careful selection of appropriate prophylactic measures and the determination of optimal timing for post-transplant HBV monitoring could be critical for improving outcomes in pediatric liver transplant recipients. Future studies should aim to refine these approaches and further elucidate the long-term clinical implications of using HBcAb-positive grafts in pediatric patients.

## Supplementary Information

Supplementary Material 1

## Data Availability

Data is available upon request to the corresponding author.
