# Supplementary material for: Risk of HBV transmission from HBcAb-positive grafts in pediatric liver transplantation: a real-world prospective cohort study
Source: Virol J. 2025 Sep 29;22:312. doi: 10.1186/s12985-025-02941-1 (PMC12482531; doi:10.1186/s12985-025-02941-1)

**Supplementary material**

**Supplementary method**

1. **ddPCR detection of HBV total DNA and cccDNA**

A 20ul ddPCR reaction mix comprised 10ul of 2× ddPCR Supermix for probes (no dUTP) (Bio-Rad, Pleasanton, California USA), 1ul of 20× primers/probe mix (900nM and 250nM) and 9ul of digested/purified DNA sample. Reaction droplets were generated according to manufacturer’s protocol by QX200TM Droplet Generator (Bio-Rad, Hercules, California, USA). Intrahepatic HBV total DNA and cccDNA was amplified using T100^TM^ Thermal Cycler (Bio-Rad, Hercules, California, USA) with the following amplification profile: an initial denaturation cycle of 10 min at 95℃, followed by 40 cycles of denaturation for 30s at 94℃, annealing for 60s at 60℃ (for HBV total DNA) and 62℃ (for HBV cccDNA), a final incubation of 10 min at 98℃ (ramp rate 2℃/s). After amplification, positive and negative droplets were quantified by a QX100TM Droplet Reader (Bio-Rad, Hercules, California, USA) using QuantaSoftTM analysis software version 1.7.4 (Bio-Rad, Hercules, California, USA). Appropriate negative and positive controls were included in each PCR experiment

**Supplementary Table**

**Supplementary Table 1.**

1. **Primers and probes used for detecting HBV total DNA and cccDNA through ddPCR assay.**

| **Primer/probe set** | **Sense primer** | **Antisense primer** | **Probe** |
| --- | --- | --- | --- |
| HBV total DNA (S region) | GTGTCTGCGGCGTTTTATCA | GACATACGGGCAACATACC | VIC-CCTCTICATCCTGCTGCTATGCCTCA-BHQ1 |
| HBV total DNA (C region) | TTCCGGAAACTACTGTTGTTAGAC | ATTGAGATTCCGAGATTGAGA | FAM-CCCTAGAAGAAGAACTCCCTCGCCTC-BHQ1 |
| HBV cccDNA | CGTCTGTGCCTTCTCATCTGC | GCACAGCTTGGAGGCTTGAA | FAM-CTGTAGGCATAAATTGGTCTGCGAA-BHQ1 |

1. **Primers used for nested PCR and sequencing.**

| **Primer set** | **Sense primer** | **Antisense primer** |
| --- | --- | --- |
| *S region* |  |  |
| S1/S2 | CATCAGGATTCCTAGGACCCCT | AGGACAAACGGGCAACATAC |
| S3/S4 | CTTGTTGACAAGAATCCTCACA | CCAACAAGAAGATGAGGCATA |
| *Pre-core/core Region* |  |  |
| C1/C2 | TCACCTCTGCCTAATCATC | GAGGGAGTTCTTCTTCTAGG |
| C3/C4 | TTCAAGCCTCCAAGCTGTGCC | AGGAGTGCGAATCCACACTCC |
| *Pol Region* |  |  |
| P1/P2 | CGTCGCAGAAGATCTCAATC | TCTTGTTCCCAAGAATATGGT |
| P3/P4 | CCTTGGACTCATAAGGT | TCCCAAGAATATGGTGACCC |
| *X Region* |  |  |
| X1/X2 | CGCCAACTTACAAGGCCTTTC | GGCGTTCACGGTGGTCTCCAT |
| X3/X4 | CCATACTGCGGAACTCCTAG | CGTAAAGAGAGGTGCGCCCC |

**Supplementary Table 2. Results of DNA concentration and GAPDH gene amplification in extracted liver tissue samples.**

| **Patient** | **Concentration of**  **extracted DNA (ng/ul)** | **Ct values of the**  **GAPDH genes** |
| --- | --- | --- |
| #2 | 479 | 12.4 |
| #3 | 218 | 14.1 |
| #5 | 462 | 12.2 |
| #6 | 311 | 12.8 |
| #7 | 447 | 12.4 |
| #9 | 409 | 12.6 |
| #10 | 288 | 13.4 |

**Supplementary table 3. Results of nested-PCR and ddPCR assay for HBV DNA and HBV cccDNA.**

| **Patient** | **Nested PCR for total HBV DNA** | | | | | **ddPCR for total HBV DNA (copies/10ul)** | **ddPCR for HBV cccDNA (copies/10ul)** |
| --- | --- | --- | --- | --- | --- | --- | --- |
|  | **S** | **Core** | **Pol** | **X** | **Score** |  |  |
| #2 | - | - | - | - | 0/4 | - | - |
| #3 | - | - | - | - | 0/4 | 8 | 2 |
| #5 | - | - | - | - | 0/4 | - | - |
| #6 | - | - | - | - | 0/4 | - | - |
| #7 | - | - | - | - | 0/4 | - | - |
| #9 | - | - | - | - | 0/4 | - | - |
| #10 | - | - | - | - | 0/4 | - | - |

**Supplementary figure 1. The level of HBsAb titer in the 10 children grouped by HBV viral load.**


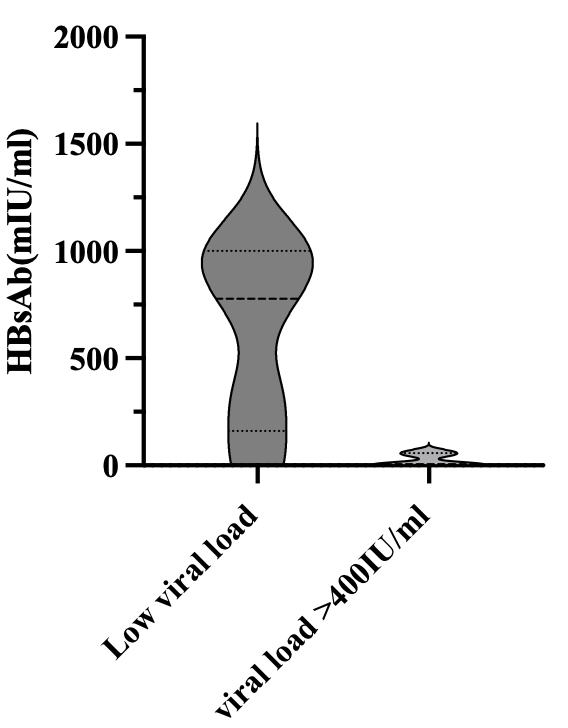


**Supplementary figure 2.**

(A) Dual-channel ddPCR detection of different gene regions in gradient-diluted HBV plasmid samples.


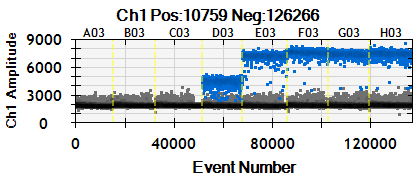

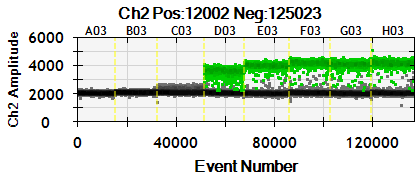


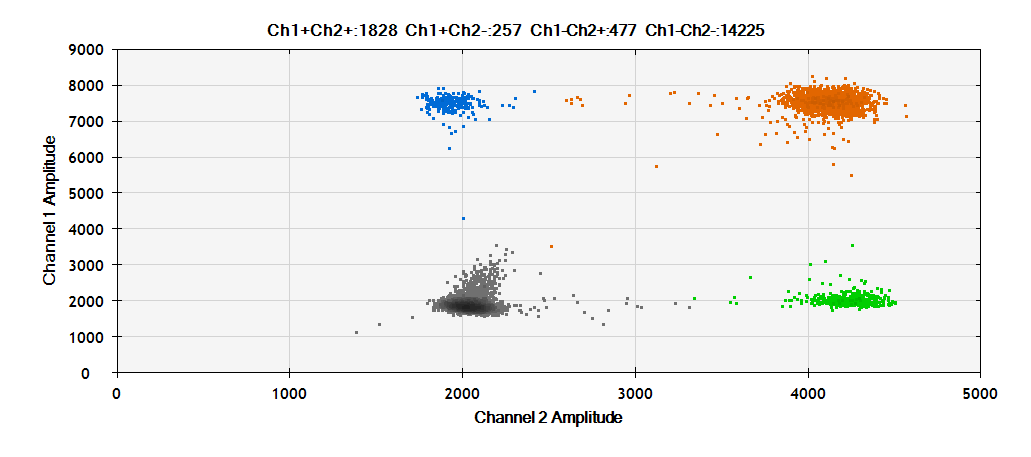


(B) PSAD treatment of HBV superhelix plasmid DNA and linearized plasmids.


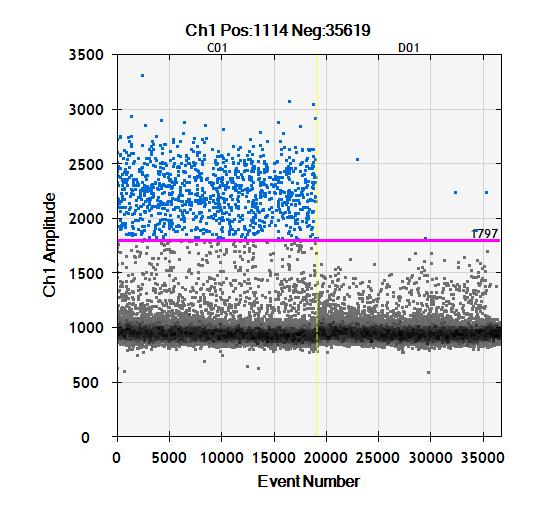

Supplement: Supplementary file 1 — Supplementary Material 1 [file 12985_2025_2941_MOESM1_ESM.docx]
